# Nonoperative Management of Gartland Type II Supracondylar Humeral Fractures: A Comprehensive Review

**DOI:** 10.1007/s12178-024-09937-4

**Published:** 2025-01-07

**Authors:** Michaela Booker, Faith Sumandea, Nirav Pandya, Ishaan Swarup

**Affiliations:** 1 San Francisco, USA; 2 Elk Grove, USA

**Keywords:** Supracondylar humeral fracture, Supracondylar fracture, Supracondylar, Humerus fracture, Gartland type 2, Gartland type II, Gartland, Pediatric fractures, Pediatric trauma, Pediatric orthopedics, Pediatric humerus fractures, Type II fractures, Type 2 fractures, Orthopedic trauma, Trauma, Nonoperative type II fracture, Operative type II fractures, Nonoperative gartland type II fracture, Operative gartland type II fracture, Nonoperative gartland fractures, Operative gartland fractures, Nonoperative gartland type 2, Operative gartland type 2, Extension type supracondylar fractures

## Abstract

**Purpose of Review:**

This review aims to provide a comprehensive analysis of the nonoperative management of Gartland Type II fractures in pediatric patients.

**Recent Findings:**

Supracondylar humeral fractures (SCF) are one of the most common traumatic fractures in pediatric populations, characterized as transverse fractures at the distal humerus between the medial and lateral columns. Early studies strongly opposed closed reduction and casting as an acceptable treatment modality for Gartland type II fractures as an early case series showed high rates of complications; however, more recent studies have suggested better outcomes.

**Summary:**

The optimal management of Gartland Type II supracondylar fractures has yet to be fully elucidated. This review highlights the indications, complications, and outcomes of nonoperative Gartland Type II supracondylar humeral fracture management. Additionally, it demonstrates the need for further research to inform guidelines on managing this condition.

## Introduction

Supracondylar humeral fractures (SCF) are one of the most common traumatic fractures that occur in pediatric populations. These injuries are characterized by transverse fracture lines located at the distal humerus between the medial and lateral columns [1]. SCFs commonly occur in pediatric populations because the bone in this area undergoes metaphyseal remodeling during early development, causing a decreased anteroposterior diameter [2, 3]. This creates an imbalance in tensile forces because the anterior capsule and ligaments are thicker and stronger than the posterior capsule within the elbow joint, making the supracondylar area susceptible to injuries, especially under extension forces.

There are several types of SCFs, with extension-type fractures being the most common [4]. Extension-type SCFs are managed with either operative or nonoperative treatment [4]. The Gartland classification is used to describe this fracture and guide clinical management. Gartland type I fractures are stable and can be managed nonoperatively, while types III and IV are unstable and treated with surgery [5]. There is a growing body of literature on managing risks and benefits of nonoperative and operative management of Gartland type II fractures. However, the preferred treatment modality remains controversial.

This review aims to provide a comprehensive analysis of supracondylar fractures, including the epidemiology, classification, and complications, and an in-depth analysis of current practices for managing Gartland Type II SCF.

## Epidemiology

Supracondylar humerus fractures are the most common type of elbow injury in children. According to a recent study using the Nationwide Emergency Department Sample database, 60 to 72 per 100,000 children present annually to the emergency department with concerns for SCF, accounting for 63,348 emergency room visits yearly [6]. Most fractures occur in children between the ages of 5 and 7, with the extension type fracture being the most common [7]. Approximately 70% of SCFs are caused by falls in children with outstretched hands, which creates instability in flexion, extension, or both, leading to a fracture [8]. Often, the primary mechanism of these elbow injuries is falling from elevated surfaces. In children under three years of age, falling from a height of less than 3 feet can lead to this type of injury, whereas in older children, greater heights, such as falling from monkey bars or swings, are often required [6, 9]. Although open fractures are less likely to occur, they are usually found amongst older children who sustain injuries with a higher impact mechanism, which are also more likely to be associated with neurovascular injuries [6]. Most cases of SCF occur during the summer months, with some studies reporting rates as high as 60% [6, 10].

## Classification of Supracondylar Fractures

**Fig. 1:**
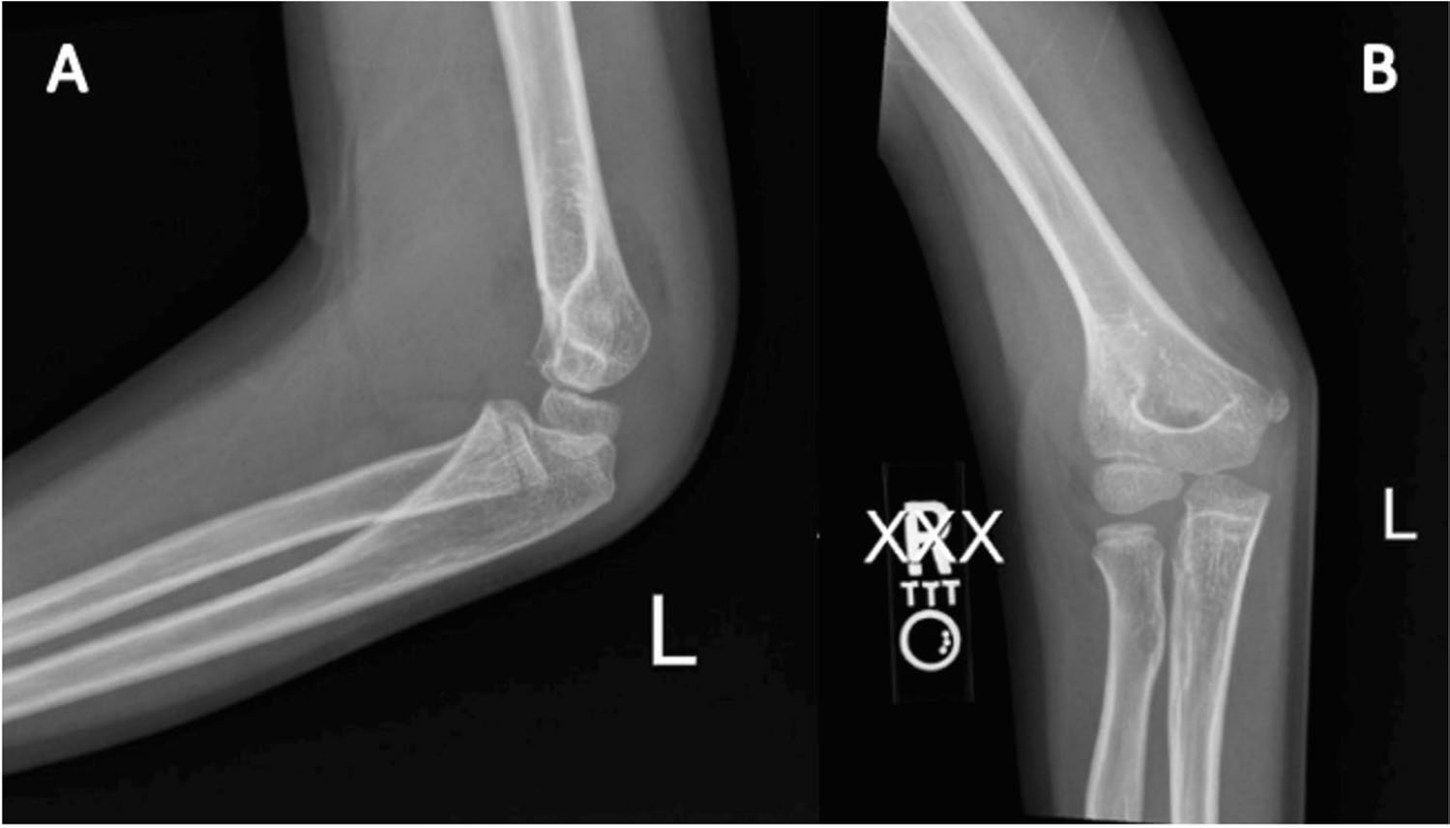
Lateral (**A**) and anterior–posterior (**B**) views of a Type 1 SCF of the left arm

**Fig. 2:**
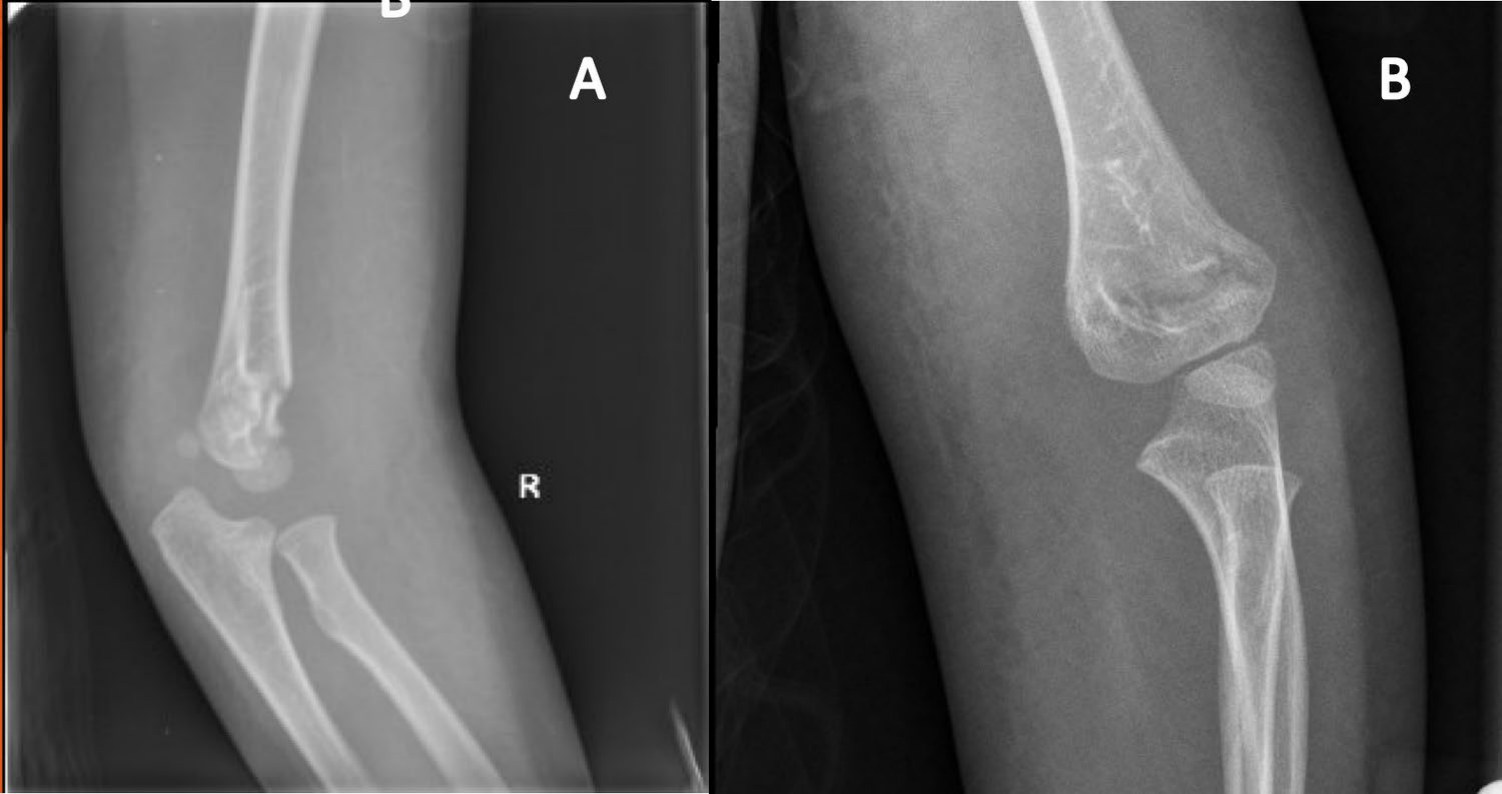
Anterior–posterior (**A**) and lateral (**B**) views of a Type 2 SCF of the right arm

**Fig. 3:**
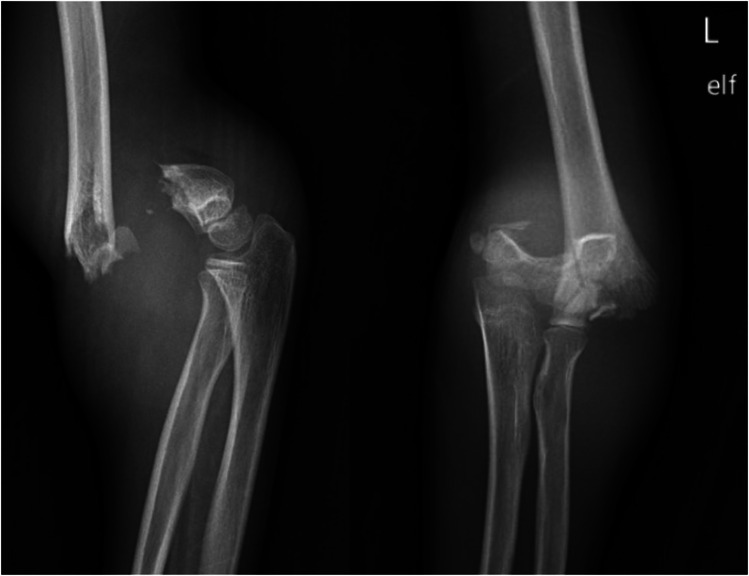
Lateral (**A**) and anterior–posterior (**B**) views of a Type 3 SCF of the left arm

There are two types of SCFs: extension type, accounting for 98% of fractures, and flexion type, accounting for 2% [2, 3, 11]. Based on several factors, SCFs are further classified into four categories depending on the degree of cortex involvement and severity of displacement [2]. The Gartland classification system defines SCF into four types, as shown in Table 1. Images of each fracture type are shown in Figs. 1, 2, and 3.

**Table 1:** Gartland fracture classification system

Gartland Type:	Gartland Type:
Type I	Nondisplaced
Type II	Displaced in one plane with posterior cortex and periosteum intact
Type III	Displaced in two or three planes
Type IV	Displaced with complete periosteal disruption and instability in flexion and extension

Type II fractures have been further delineated into type IIa and type IIb, classified by the Wilkins modification [1, 11]. Type IIA fractures are characterized by displacement without malrotation, whereas Type IIb fractures are characterized by extension and malrotation.

In recent years, numerous studies and review articles have outlined the most common treatment practices determined by their Gartland types. Type I SCF are commonly treated nonoperatively, while types III and IV undergo operative treatment with closed or open reduction and percutaneous pinning (CRPP or ORPP) [12]. The treatment of type II SCF remains controversial. Some orthopedic surgeons recommend surgical management, while others advocate for nonoperative management [1, 2, 11]. In either case, there is a risk of extension deformity of the elbow, which may result in a lack of elbow flexion and functional limitations. Some studies suggest that remodeling may occur in the elbow, especially in children under the age of 8 [13].

## Treatment of Supracondylar Fractures

### Surgical Management of Type II SCF

**Fig. 4:**
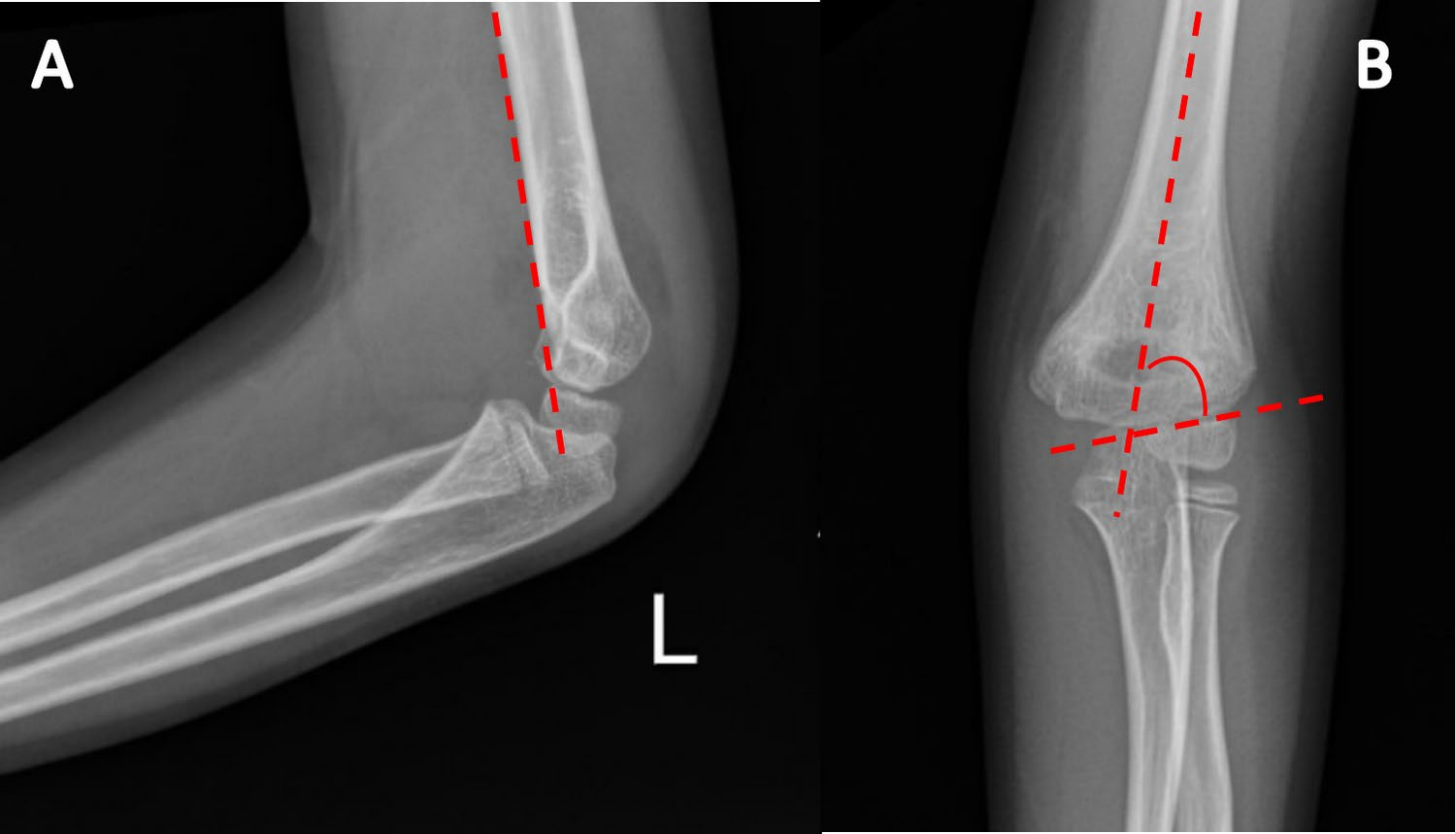
Illustrates the anterior humeral line (**A**) and the Baumann angle (**B**)

In general, type II SCFs are most commonly treated with CRPP [3]. This consists of a closed reduction under anesthesia followed by percutaneous fixation with smooth Kirschner wires. This allows for correcting the extension deformity, and the pins hold the reduction to minimize re-displacement. Radiographically, the normal alignment of the humerus to the elbow joint can be achieved by re-establishing the anterior humeral line and the Baumann angle (Fig. 4). The anterior humeral line should pass through the capitellum, and the Baumann angle should measure 64–81º [14].

Then, a cast is applied. Pins are usually removed in the outpatient setting at 3–4 weeks, during which patients may begin ranging the elbow [2, 3, 15].

The risks of surgical management include anesthetic risks, neurovascular injury caused by the pins, and pin site infection [16]. The risk of a severe complication from anesthesia is less than 1% and includes aspiration, medication reactions, pulmonary complications, the requirement of blood transfusions, postoperative cognitive dysfunction, and postoperative nausea and vomiting [17, 18]. Because of these risks, there is an increase in the use of nerve-block anesthesia and local infiltration anesthesia [19]. Although these risks are small, they can be devastating. In addition to the anesthetic risks of CRPP, pins can also lead to neurovascular injuries. Medial pins with lateral pin start points have an increased risk of ulnar nerve injury, with some studies demonstrating that over half of their ulnar injuries are medial pin placement alone [20, 21]. The rate of pin infection is approximately 1%, and most are reported to be superficial and may be caused by hypergranulation tissue, pin migration, and loosening or bundling of pin tract irritation [22, 23]. Pin site infections are often treated with antibiotics and wound care [24]. Although the risks are minimal and easily treatable, there are rare instances where deep infections can occur, which would necessitate more aggressive therapies, including hospitalization, intravenous antibiotics, and surgical debridement [22].

### Indications for Non-Operative Management

Some type IIa fractures can be treated nonoperatively. In a study by Pierantoni et al., indications for nonoperative management included displacement of 2–15 mm or a 15 – 20º extension on the lateral view and a partially intact posterior cortex, which can maintain reduction without fixation [8]. Similarly, in a study by Spencer et al., indications for nonoperative management of type II fractures included fractures with minimal rotational deformities, no coronal malalignment, and no significant extension of the distal fragment [25].

These fractures typically undergo closed reduction under conscious sedation in the emergency room, followed by long arm casting. A closed reduction maneuver is performed in which a flexion and an anteriorly directed force are exerted on the distal fragment, with an additional posteriorly directed force on the distal humerus if necessary [26]. After reduction, alignment can be confirmed using fluoroscopy. Casting is performed by applying a well-molded long-arm cast to the forearm held in pronation and the elbow flexed at 80–85º [8]. The cast is usually in place for 4–5 weeks to maintain healing and alignment. For patients who may not be able to follow up closely, CRPP should be considered.

The risks of nonoperative management include the inherent risk of sedation, such as respiratory depression, hypotension, allergic reactions, etc. Additionally, while conscious sedation may provide certain benefits, such as a decreased time to manipulation and shorter length of stay, the risk of loss of reduction may be elevated [27]. Therefore, close and frequent follow-up is required to ensure appropriate fracture alignment is maintained.

### Outcomes

Several studies have assessed the outcomes of nonoperative management of type II supracondylar humerus fracture, some of which have estimated that nonoperative management’s failure rate is around 20–25% [27, 28]. A study by Spencer et al. suggested that up to 77% of patients undergo unnecessary surgical procedures [25]. They conducted a retrospective analysis of 259 patients with type II SCF and compared operative and nonoperative outcomes. They found that with appropriate follow-up, those treated nonoperatively had similar outcomes to those with surgical fixation.

Flynn’s criteria is a widely used tool to assess functional outcomes of supracondylar humeral fracture, combining range of motion and cosmetic outcomes such as changes in carrying angles [29]. Perantoni et al. reported excellent outcomes in 8 (25.8%) patients, suitable in 14 (41.9%), fair in 4 (12.9%), and poor in 1 (3.2%) [30]. Other studies reported similar findings, with most functional outcomes being either excellent or good [5, 12, 25]. Another tool utilized in evaluating functional outcomes is the shortened version of the Disabilities of the Arm, Shoulder, and Elbow questionnaire (Quick DASH). The Quick DASH is a self-reported questionnaire comprising 11 items related to various symptoms and activities of daily living that patients may experience [30]. Scores range from 0 (most minor disability) to 100 (most disability). Pierantoni et al. reported Quick DASH scores of 22.4 at cast removal (range: 19–40) and 2.3 (range: 0–9) at the last follow-up visit (P < 0.001) in patients without secondary displacement [30].

### Risk Factors for Failure of Nonoperative Management

Several studies have investigated risk factors associated with increased rates of failure of conservative treatment to help stratify which patients may benefit from operative management. Spencer et al. found that initial rotational deformity, coronal malalignment, and significant extension of the distal fragment are associated with an increased rate of nonoperative treatment failure [27]. Fitzgibbons et al. reported similar discoveries, finding that a more substantial extension of the distal humeral fragment at the time of injury was associated with increased failure rates [31]. Additionally, Ojeaga et al. reported that lower humerocondylar angles at presentation and failure to recreate the distal humerus “hourglass” and perpendicular distance from the anterior humeral line to the capitellum were associated with increased rates of failure [2]. Lastly, patients who may have issues with cast immobilization and adherence to restrictions, such as behavioral issues or developmental delay, may be at risk for failure with nonoperative management. [32]

### Complications

Some of the most feared complications associated with nonoperative management of Gartland type II fractures are compartment syndrome and Volkmann’s ischemic contractures. Early studies strongly opposed closed reduction and casting as an acceptable treatment modality for Gartland type II fractures, as an early case series showed high Volkmann ischemic contractures and cubitus varus deformities [30, 33]. However, more recent studies estimate the rate of compartment syndrome or Volkmann’s ischemia to be < 1% [30]. Other potential complications include loss of reduction, fishtail deformity of the distal humerus, cubitus valgus, cubitus varus, and anterior interosseous nerve palsy [2, 5, 27, 30]. Malunions may require subsequent surgical management, consisting of a distal humerus osteotomy [34]. Indications of distal humerus osteotomy include pain or functional limitations after fracture healing and skeletal maturity [34].

## Conclusion

Gartland Type II fractures are relatively common in the pediatric population. These injuries are traditionally managed operatively with CRPP; however, some type IIa fractures may be amenable to closed reduction and casting. There is a risk for re-displacement and failure with nonoperative management, and risk factors include substantial initial rotational deformity, coronal malalignment, and more significant extension of the distal humeral fragment. Additionally, there are risks to surgical management, which include anesthesia risks, pin site infections, and neurovascular injuries from pin application. Patients and families should be appropriately counseled about the risks and benefits of both approaches, and treatment decisions should be made on a case-by-case basis.

## Data Availability

No datasets were generated or analysed during the current study.
