# Correction: Nonoperative Management of Gartland Type II Supracondylar Humeral Fractures: A Comprehensive Review

**DOI:** 10.1007/s12178-025-09959-6

**Published:** 2025-03-12

**Authors:** Michaela Booker, Faith Sumandea, Nirav Pandya, Ishaan Swarup

**Affiliations:** 1 San Francisco, USA; 2 Elk Grove, USA

**Correction to: Current Reviews in Musculoskeletal Medicine (2025) 18:48–53**

10.1007/s12178-024-09937-4

In this article, the order in which the authors appeared in the author list was incorrectly given as “Michaela Booker · Faith Sumandea · Ishaan Swarup · Nirav Pandya” where it should have been “Michaela Booker · Faith Sumandea · Nirav Pandya · Ishaan Swarup”.

The original article has been corrected.

